# SARS-CoV-2 Infection and Significance of Oral Health Management in the Era of “the New Normal with COVID-19”

**DOI:** 10.3390/ijms22126527

**Published:** 2021-06-18

**Authors:** Kenichi Imai, Hajime Tanaka

**Affiliations:** Department of Microbiology, School of Dentistry, Nihon University, Chiyoda-ku, Tokyo 101-8310, Japan; tanaka.hajime@nihon-u.ac.jp

**Keywords:** COVID-19, SARS-CoV-2, oral bacteria, oral health, saliva

## Abstract

More than a year ago, the coronavirus disease 2019 (COVID-19), caused by severe acute respiratory syndrome coronavirus 2 (SARS-CoV-2), was declared a pandemic by the World Health Organization, with the world approaching its fourth wave. During this period, vaccines were developed in a much shorter period than thought possible, with the initiation of the pertinent vaccination. However, oral cavities have come under renewed scrutiny worldwide because saliva, a mixture of salivary secretions, pharyngeal secretions, and gingival crevicular fluid, have not only been shown to contain infective viral loads, mediating the route of SARS-CoV-2 transmission via droplet, aerosol, or contagion, but also used as a sample for viral RNA testing with a usefulness comparable to the nasopharyngeal swab. The oral cavity is an important portal for ingress of SARS-CoV-2, being an entryway to the bronchi, alveoli, and rest of the lower respiratory tract, causing inflammation by viral infection. Moreover, angiotensin-converting enzyme 2, a host receptor for SARS-CoV-2, coupled with proteases responsible for viral entry have been found to be expressed on the tongue and other oral mucosae, suggesting that the oral cavity is the site of virus replication and propagation. Furthermore, there is a possibility that the aspiration of oral bacteria (such as periodontal pathogens) along with saliva into the lower respiratory tract may be a complicating factor for COVID-19 because chronic obstructive pulmonary disease and diabetes are known COVID-19 comorbidities with a greater risk of disease aggravation and higher death rate. These comorbidities have a strong connection to chronic periodontitis and periodontal pathogens, and an oral health management is an effective measure to prevent these comorbidities. In addition, oral bacteria, particularly periodontal pathogens, could be proinflammatory stimulants to respiratory epithelia upon its exposure to aspirated bacteria. Therefore, it may be expected that oral health management not only prevents comorbidities involved in aggravating COVID-19 but also has an effect against COVID-19 progression. This review discusses the significance of oral health management in SARS-CoV-2 infection in the era of “the new normal with COVID-19” and COVID-19 prevention with reference to the hypothetical mechanisms that the authors and the other researchers have proposed.

## 1. Introduction

More than a year since the coronavirus disease 2019 (COVID-19) was declared a pandemic by the World Health Organization (WHO), the eagerly awaited vaccination has finally begun. According to a statement from WHO, by the end of April 2021, >146 million people had been infected with severe acute respiratory syndrome coronavirus 2 (SARS-CoV-2), with 3.09 million deaths [1].

Fortunately, several vaccines, based either on new or prior production systems, have been developed in a much shorter period than had been thought possible with the aim of specifically blocking the attachment of viruses to host receptors. However, the increase rate in the number of infected individuals remains high due to lacking relevant medicine, insufficient production and delivery of vaccines, and the emergence of mutant strains that may negate the effectiveness of these newly developed vaccines.

It is no exaggeration to state that the history of medical progress has been the history of the fight against infectious diseases. In fact, pandemics have recurred throughout human history. For example, the plague, which was spread by the Mongolian army through their expedition to Europe, prevailed between 1347 and 1352, killing approximately 30 million people in Europe alone, which was equivalent to 40% of the European population at that time. Meanwhile, the Spanish flu that mostly infected the US Army was spread worldwide from 1918 to 1920, as US troops were deployed to Europe and Africa. The death toll was estimated to be approximately 40 million, and the number of patients reached 500 million, which was close to 30% of the world population at that time. The high number of deaths recorded because of the flu was determined to be due to pneumonia caused by secondary bacterial infections [2].

One important factor worsening the pandemic is the movement of people, which has been accelerated thanks to the development of modern transportation, allowing the spread of the infection in densely populated environments, often called as the “three Cs”. The latter is composed of “closed spaces with poor ventilation”, “crowded places with many people nearby”, and “close-contact setting such as close-range conversations”.

The oral cavity is a key gateway for viral invasion, being one of the first places where the virus interacts with the host. In addition, the oral cavity is home to an abundant and unique microbial flora, such as bacteria and viruses, which can disseminate through the blood stream and salivary flow to the whole body. Therefore, it is important to consider infections caused by the oral flora not only as diseases localized within the oral cavity but also as diseases affecting the whole body. These oral bacteria, such as periodontal pathogens, may accelerate viral infectious diseases such as COVID-19.

Recent scientific findings from this point of view have shown that the oral mucosa can be infected with SARS-CoV-2 and that saliva can act as a high-quality source similar to the nasopharyngeal swab to diagnose COVID-19-infected individuals. The findings have also described that infected individuals with respiratory diseases, such as chronic obstructive pulmonary disease (COPD) or pneumonia, and with comorbidities, such as diabetes, are more likely to aggravate and consequently have a higher mortality rate. Notably, both diseases have been linked to the presence of chronic periodontitis.

Thanks to the numerous research reports published in a short period of time, the outline of SARS-CoV-2/COVID-19 is rapidly being clarified. In this review, we will introduce the relationship between SARS-CoV-2 infection and the oral cavity and further discuss the significance of oral health management in SARS-CoV-2 infection focusing on the newly published research results, including those by the authors in the past year.

## 2. A New Coronavirus SARS-Cov-2 and Its Infection COVID-19

Four coronaviruses, i.e., 229E, OC43, NL63, and HKU-1, have been known to cause 10%–15% of the common cold syndromes in humans. In the last two decades, two new human pathogenic coronaviruses have been discovered: one is SARS-CoV-1, the pathogen responsible for the SARS epidemic that raged across China from 2002 to 2003; the other is MERS-CoV, the pathogen responsible for the Middle East respiratory syndrome (MERS) that spread mainly in the Middle East and South Korea in 2012 (Table 1).

SARS-CoV-2 has been determined to be approximately 80% genetically identical to SARS-CoV-1 and approximately 90% genetically identical to bat CoV 2 [3], which thus led the International Committee for the Classification of Viruses to name the new coronavirus as SARS-CoV-2. In addition, on February 11, 2020, the WHO has referred to this infectious disease caused by SARS-CoV-2 as COVID-19.

**Figure 1:**
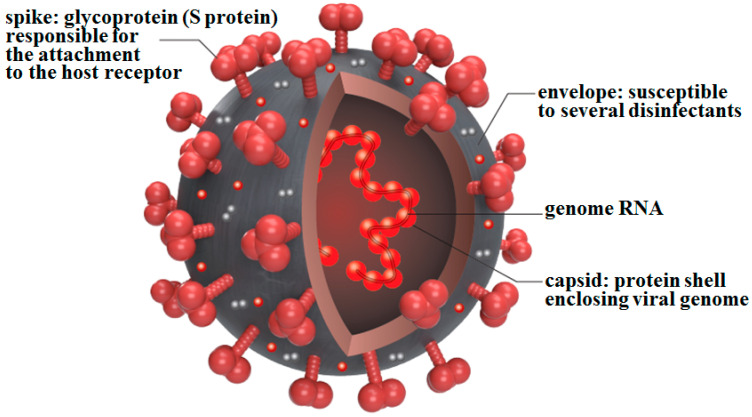
**Schematic diagram of SARS-CoV-2.** SARS-CoV-2 has a simple structure consisting of a capsid that covers genomic RNA and an envelope that covers the capsid. The envelope is pierced by hundreds of spikes composed of S protein. The S protein has been used as an antigen for all newly developed vaccines.

The structure of the SARS-CoV-2 viral particle, like that of other coronaviruses, is a simple single-stranded RNA genome covered by a protein capsid and a lipid bilayer called an envelope (Figure 1).

The SARS-CoV-2 genome is approximately 30 kb (30,000 bases) in size, the largest of any RNA virus; this is three times larger than that of the influenza virus (approximately 10 kb), which is recognized as having a relatively large genome among the RNA viruses. In addition, because of its large genome, SARS-CoV-2 can express enzymes that repair its own genome in a similar manner to that seen in bacteria. The viral envelope is pierced by hundreds of spike (S) proteins, which are vital in the adsorption to the target cell and subsequent infection. On the other hand, SARS-CoV-2 remains susceptible to many disinfectants, as it loses its infectivity when in contact with disinfectants.

To prevent SARS-CoV-2, contact, droplet, and aerosol transmissions should be avoided. SARS-CoV-2 can be transmitted from asymptomatic subjects; therefore, it is important to wear masks, disinfect hands and fingers, ventilate the area, and avoid the “three Cs” (confined spaces, crowded places, and close-contact environments). The basic reproduction number (R0, how many people are infected by one infected person) of SARS-CoV-2 has been determined to be initially between 1.4 and 2.5; however, recently, it has been reported to have an average of 3.28. This means that SARS-CoV-2 is more infectious than the H1N1 novel influenza viruses as the latter only had R0 values of 2.0 since 2009.

**Figure 2:**
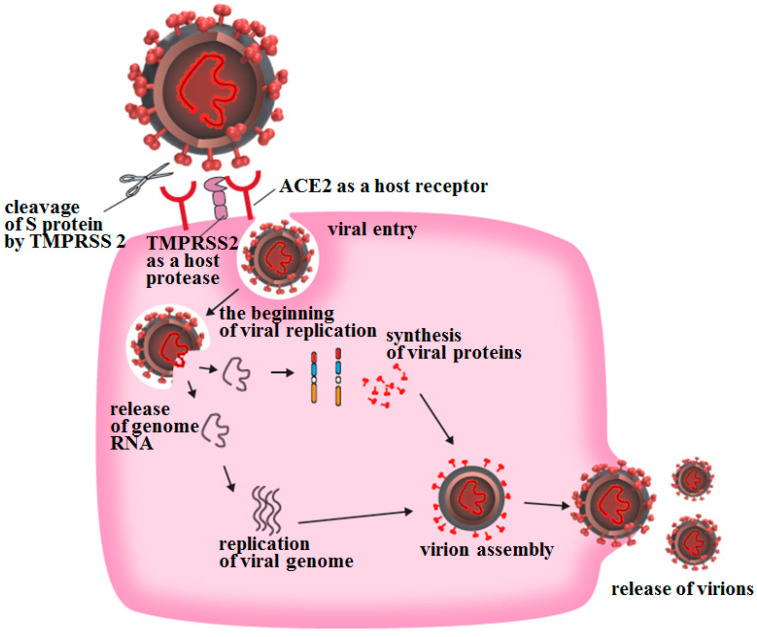
**Adsorption and entry of SARS-CoV-2 into host cells.** SARS-CoV-2 begins to invade target cells when the viral S protein expressed on the envelope binds to the ACE2 receptor, which is often expressed on the cytoplasmic membrane of target cells.

SARS-CoV-2 infection of target cells begins when the S protein binds to angiotensin-converting enzyme 2 (ACE2) expressed on the host cell surface (Figure 2). The S protein is then cleaved by the proteolytic activity of transmembrane protease, serine 2 (TMPRSS2), a serine protease expressed on the cytoplasmic membrane of the target cell, and membrane fusion between the envelope and the cytoplasmic membrane is initiated [4]. The S protein contains a sequence that can be cleaved by another serine protease secreted by human cells called furin, and it has been suggested that proteolytic activity of furin may also promote this membrane fusion.

**Figure 3:**
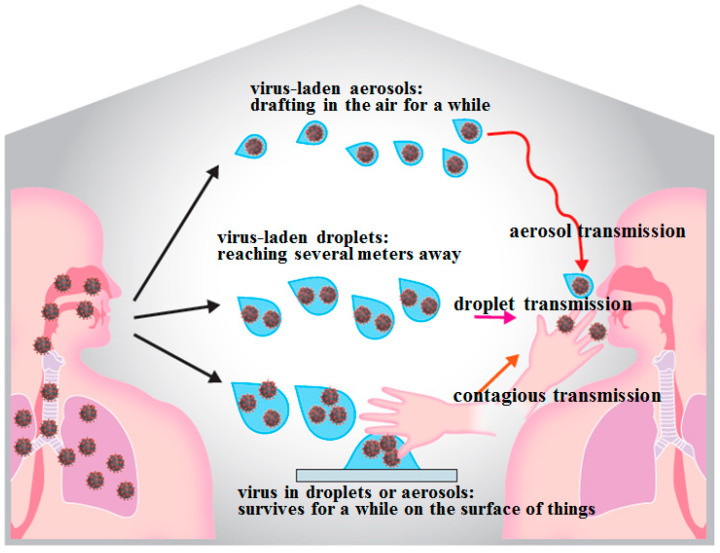
**Mode of transmission of SARS-CoV-2**. To prevent SARS-CoV-2 infection, contact, droplet, and aerosol transmissions should be avoided. SARS-CoV-2 can be transmitted from asymptomatic subjects; therefore, it is important to wear masks, disinfect hands and fingers, ventilate the area, and avoid the “three Cs” (confined spaces, crowded places, and close-contact environments).

Among the known routes of SARS-CoV-2 infection, droplet and contact infections are the most important and practical ones (Figure 3). The virus has been determined to survive on cardboard for up to 24 h, on copper for up to 4 h, and on stainless steel and plastic for up to 2–3 days [5]. SARS-CoV-2 is more stable in the environment than influenza viruses; thus, frequent and careful hand washing and disinfecting are important to prevent infection. SARS-CoV-2 can also survive in aerosols for 3 h [5]; hence, being in an enclosed space with an infected person can lead to aerosol infection, which is an intermediate mode of infection between droplet infection and droplet-nuclei infection. Therefore, in dental treatments where aerosols are frequently generated, high-level preventative measures are required to avoid the generation and spread of aerosols (see below for details).

More importantly, unlike SARS-CoV-1 infection, asymptomatic SARS-CoV-2-infected persons can spread the virus even before the onset of pneumonia; this is the so-called “incubation phase infection”, which only makes infection prevention measures difficult. Reports indicate that some infected but asymptomatic persons have pneumonia as per chest imaging findings [6] and that 59% of asymptomatic individuals are infecting others at this stage [7]. Therefore, “maintaining social distancing”, or, in other words, “avoiding the three Cs (confined spaces, crowded places, and close-contact environments)”, is of utmost importance in preventing its spread. As dentistry is an aerosol-producing profession, frequent ventilation of examination rooms and gargling by patients and medical staff, as well as hand disinfection, are deemed effective measures to prevent the outbreak of nosocomial infections or “disease cluster formation” in dental practices.

The clinical manifestations of COVID-19 develop after an incubation period of approximately 5 days, and these include fever, fatigue, dry cough, myalgia, and sore throat. As the inflammation spreads throughout the lungs, blood oxygen levels decrease. In addition, severe respiratory insufficiency, such as acute respiratory distress syndrome (ARDS), increases the mortality rate. Elderly patients with COVID-19 and patients with COVID-19 and comorbidities, such as COPD, diabetes, and cardiovascular disease, are known to be more susceptible to severe disease and mortality.

The involvement of the cytokine storm has been pointed out as a major factor in ARDS. Elevated levels of proinflammatory cytokines, such as IL-6, are associated with increased mortality, suggesting that excessive inflammation contributes to poor prognosis [8,9,10]. In addition to respiratory insufficiency, COVID-19 may be associated with other complications, such as infarction following thrombus formation, myocardial dysfunction, arrhythmias, acute kidney injury, and gastrointestinal disorders.

## 3. Relationship between SARS-CoV-2 and the Oral Cavity

As will be discussed below, relatively large amounts of SARS-CoV-2 receptors have been found to be expressed on the oral mucosae, including the tongue. Therefore, saliva together with nasopharyngeal swabs can be used for COVID-19 testing. The significance of the oral cavity in COVID-19 diagnosis has, hence, attracted widespread attention from the medical community. In viral infections, such as influenza and human immunodeficiency virus infections, the involvement of oral bacteria in the development and progression of these infections has been highlighted [11,12,13,14]. From these perspectives, it is, therefore, assumed that the prevention and improvement of an underlying disease via oral health management will be an important means of preventing the onset and progression of COVID-19. First, details of the oral cavity will be discussed below.

### 3.1. Oral Symptoms Of COVID-19 Patients ans the Significance of ACE2 Expression in the Oral Cavity

**Figure 4:**
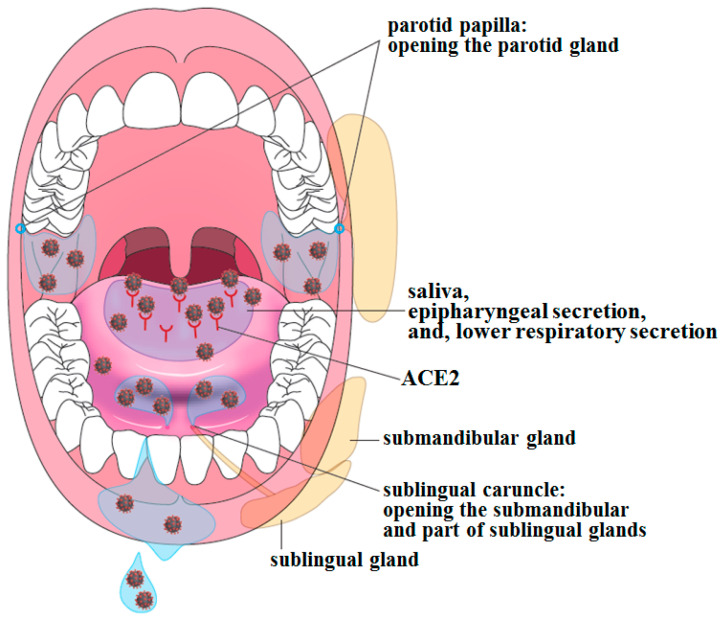
**Relationship between ACE2 expression in the oral cavity and SARS-CoV-2 being detected in saliva.** ACE2 is expressed on the oral mucosae, particularly on the dorsum of the tongue and on the gingiva, suggesting that the oral mucosae can be infected with SARS-CoV-2 and that oral bacteria present may interact with SARS-CoV-2. The infection of salivary glands themselves with SARS-CoV-2 has recently been reported [18]. A myriad of viruses has been detected in saliva, and this has led to measures on how to prevent droplet and aerosol transmissions. This also means that saliva can be used as a specimen for testing viral infection.

Although the number of studies examining the relationship between the oral cavity and SARS-CoV-2/COVID-19 remains limited, the nature of this interaction is gradually becoming clear. It is well known that the initial symptoms of COVID-19 include taste and smell disorders. In the case of taste disorder, SARS-CoV-2 may infect cells expressing ACE2 in the tongue mucosa and then spread to adjacent taste buds, resulting in impairment of taste cell function (Figure 4).

A global search on the public databases of ACE2 mRNA expression level in human tissues shows that its expression level is relatively high in the tongue among oral mucosae, although to a lesser extent in the oral cavity than in the lungs and intestinal tract [15]. In addition, protein expression of ACE2 and TMPRSS2 has been confirmed by immunostaining in the stratified squamous epithelium of the dorsal tongue and gingiva in healthy subjects [16]. Moreover, furin, which is mentioned above, is mainly localized in the lower layers of the oral squamous epithelium and, like TMPRSS2, has also been found in saliva.

Incidentally, a meta-analysis summarizing 40 studies involving 10,228 participants from 19 countries has reported that taste disorder (45%) is the most common oral symptom, with an odds ratio of 12.68 [17]. The degree of taste disorder varies, with 38% of patients having abnormal taste, 35% having blurred taste, and 24% having loss of taste [18]. More importantly, viral load correlates with the loss of taste [18]. The authors attended the examination of suspected COVID-19 contacts in an outpatient fever clinic, wherein they were able to determine firsthand that a high percentage of those who complained of taste disorder were subsequently found to be infected with SARS-CoV-2.

Other oral clinical manifestations of COVID-19 include ulcers, vesicles, vesicular bleeding, and oral candidiasis, which have been reported to involve the mucosae of the tongue, palate, lips, gums, and cheeks [17,19,20]. However, it remains unclear whether these symptoms are caused by SARS-CoV-2 infection, a secondary phenomenon due to immune dysfunction associated with SARS-CoV-2 infection, or a superinfection with other microorganisms.

In the nasal cavity, ACE2 is expressed in the supporting cells of the olfactory epithelium, which is located just below the olfactory bulb [21], suggesting that SARS-CoV-2 infection of the olfactory epithelium can cause olfactory disorders. There have also been reports of cases where the olfactory fissure is closed due to inflammation despite normal findings in the nasal cavity [22]. Since the oral and nasal cavities are both portals of viral entry, it is possible that infection may have occurred in these areas prior to that in the lower respiratory tract.

Considering the high probability of transmission from asymptomatic patients, it is necessary for dental care providers to pay close attention to any complaints of taste or smell disorders from patients during the dental interview from the viewpoint of preventing nosocomial infections.

### 3.2. Origin of SARS-CoV-2 Detected in Saliva

As SARS-CoV-2 infection can be caused by direct inhalation of saliva droplets produced during meals, conversation, or singing, or by eating the droplets attached to food, saliva that is not contaminated by secretions from the nasal cavity or lower respiratory tract, i.e., intact saliva freshly secreted from salivary ducts, can also be a source of infection. In fact, ACE2 has been reported to be expressed in the salivary glands of patients with COVID-19, and SARS-CoV-2 has been detected in intact saliva directly collected from the opening of the submandibular gland duct, i.e., that of the sublingual peduncle [23].

Although ACE2 has also been reported, irrespective of SARS-CoV-2 infection, to be expressed in several types of cells in the ducts and acini of the minor salivary glands [18], as well as in the minor salivary gland cells of the lips and epithelial cells in the duct of submandibular glands [24], the relationship between ACE2 expression and SARS-CoV-2 infectivity is yet to be examined.

The abovementioned paper [18] has also reported that saliva from patients with COVID-19 contains epithelial cells that are both positive for ACE2 and viral RNA and that saliva collected from patients after complete recovery contains anti-SARS-CoV-2 antibodies. In another report, SARS-CoV-2 was detected in paraffin-embedded specimens obtained from pre-symptomatic patients who were later confirmed to have COVID-19, where SARS-CoV-2 RNA was detected by polymerase chain reaction (PCR) in tumor lesions on the tongue and in submandibular gland specimens [25]. Thus, SARS-CoV-2 may have infected the oral tissues during the early asymptomatic stage.

These findings suggest that the oral cavity is the site of SARS-CoV-2 infection and that saliva is the medium of SARS-CoV-2 transmission. However, important questions remain unanswered, such as whether SARS-CoV-2 replicates in the oral mucosal epithelium, including the tongue and salivary gland epithelia, and from which planktonic viruses detected in the saliva are mainly derived.

The results of the SARS-CoV-2 study in the oral cavity are eagerly awaited.

### 3.3. Usefulness of Saliva in COVID-19 Testing

The expansion of pathogen testing is clearly vital to stop the spread of infection. However, the collection of nasopharyngeal swabs for PCR testing not only requires special collecting equipment and personal protective equipment, but also creates a risk of exposure of healthcare workers to droplets emitted by the examinees; and as whoever has been tested may recall, the subjects themselves also experience distress, albeit briefly.

Since SARS-CoV-2 can be transmitted from asymptomatic subjects, it is necessary to test not only symptomatic subjects but also close contacts who are asymptomatic as early as possible. In addition, it would be difficult to use nasopharyngeal swabs in terms of the number of subjects that can be handled per unit of time when quarantining at an airport, inspecting many people in hospitals or nursing homes, or when multiple disease clusters occur.

Moreover, it has become clear that the amount of SARS-CoV-2 shed into saliva by infected individuals is comparable with that in nasal mucus. The sensitivity and specificity for virus detection in PCR tests have high agreement with those obtained with nasopharyngeal swabs, indicating that saliva is a useful material for detecting infected individuals [26,27]. Since saliva can be easily collected by the subject, and without any pain for the examinee as described above, this greatly reduces the risk of infection and labor for the healthcare worker. A few specific examples to support this are presented below.

An interesting result was reported by a group at the Department of Hematology, Hokkaido University [28]. A total of 10 confirmed patients with COVID-19 and 66 suspected patients were asked to self-collect saliva for PCR testing. The median age of the patients with COVID-19 was 69 years, the median date of specimen collection was 9 days after onset, and most of the patients’ symptoms were reported as mild to moderate. The test results showed that the virus was detected in all saliva samples collected within 2 weeks of the onset of symptoms, with a high concordance rate of 97.4% with the nasopharyngeal swabs test. The virus levels in nasopharyngeal swabs and saliva did not significantly differ, averaging 5.4 ± 2.4 and 4.1 ± 1.4 log10 copies/mL, respectively. The viral load in saliva reached a peak at the onset of the disease, was highest in the first week, and then decreased over time; the viral load in saliva decreased faster than that in nasopharyngeal swabs during convalescence.

The same group has published another study that showed the reliability of saliva testing using saliva collected from up to 1924 asymptomatic close contacts who participated in a public health department’s follow-up survey and as airport-quarantined travelers [29]. According to the report, the sensitivity (the rate of identifying true positive results) for nasopharyngeal swabs and saliva was 86% and 92%, respectively, and the specificity (the rate of identifying true negative results) for both tests exceeded 99.9%.

In addition, a paper published in the *New England Journal of Medicine* reported that saliva as a specimen can be used as a substitute to nasopharyngeal swab [30]. Notably, the amount of SARS-CoV-2 RNA on the 10th day after diagnosis was higher in saliva collected by the patients in comparison with that from nasopharyngeal swabs collected by medical personnel; the positivity rate was also higher in the self-collected samples than in the nasopharyngeal swabs. More importantly, the fluctuation of viral RNA levels was smaller in saliva than that in nasopharyngeal swabs, suggesting that saliva is a more stable sample as it is less sensitive to the collection method used.

Based on the results of these studies, PCR and quantitative antigen tests using saliva have been performed on airport quarantine subjects and close contacts; this testing has made a significant contribution to the early detection of SARS-CoV-2-infected individuals and the prevention of SARS-CoV-2 transmission. However, RNA-degrading enzymes are more abundant in saliva, and the test results may differ depending on how the collected saliva is stored and handled and which part of the viral RNA genome is targeted for PCR.

Saliva has become the main specimen of interest in developing tests for the detection of SARS-CoV-2-infected individuals. Moreover, saliva may also be an important specimen for the identification of pathogens of other viral and bacterial systemic infections. Therefore, further development of saliva-based research and data based on dental medicine are highly anticipated.

## 4. Relationship between COVID-19 Severity and Oral Bacteria: Mechanisms of Pneumonia Exacerbation

Recently, it has become clear that oral bacteria, including periodontal pathogens, are deeply involved in the onset and exacerbation of respiratory diseases, such as aspiration pneumonia, influenza, and COPD, as mentioned above. These bacteria have been detected in bronchoalveolar lavage fluid and sputum of patients with pneumonia and COPD, and the risks of developing pneumonia and COPD and the pertinent death are increased in patients with severe periodontal disease.

Therefore, studies that focus solely on the oral cavity or the pathogenicity of a single microorganism are unlikely to provide a complete picture of many infectious diseases. Thus, it is important to consider the oral cavity as a gateway to the respiratory and digestive systems and as a part of these systems.

Although there have been only few reports of bacterial superinfection or secondary bacterial pneumonia occurring in patients with COVID-19, bacteria such as *Pseudomonas aeruginosa* and *Klebsiella pneumoniae*, which are causative agents of pneumonia, have been detected in bronchoalveolar lavage fluid and sputum from infected patients, along with SARS-CoV-2 [31,32]. Additionally, patients with COVID-19 who undergo invasive ventilation for ARDS are found to be superinfected with the other causative agents of pneumonia, i.e., *Staphylococcus aureus*, *Haemophilus influenzae*, or *Streptococcus pneumoniae* [33]. The presence of elevated neutrophil counts in patients with aggravated COVID-19 and the fact that antibiotics are administered to many patients with SARS-CoV-2 also raise the possibility of secondary bacterial infection in COVID-19 aggravation. Interestingly, the detection of oral bacteria, such as *Veillonella*, *Prevotella*, *Campylobacter, Treponema*, and *Fusobacterium*, in the bronchoalveolar lavage fluid of patients with COVID-19 has been recently reported [34,35,36].

Based on the hypothesis that interactions among host-virus-bacteria may play an important role in the onset and progression of COVID-19, as they have done in influenza, the possibility of exacerbation of lower respiratory tract inflammation by aspiration of oral bacteria associated with poor oral hygiene in patients with COVID-19 will be discussed below. This is particularly relevant in the elderly and the sick who are known to be more susceptible to severe COVID-19. This is because the probability of aspiration is higher in these people due to their reduced swallowing and cough reflex function. These individuals are more likely to require more meticulous oral health management. Although the following may not be necessarily limited to SARS-CoV-2 infection/COVID-19, as it can be a common phenomenon in the lower respiratory tract inflammation associated with aspiration, we have extended our previous findings to speculate whether some aspirated oral bacteria may have enabled the establishment of SARS-CoV-2 infection or have exacerbated mild COVID-19 via the following mechanisms [37].

In addition, a retrospective case-control study has been recently published. This study assessed 568 patients with COVID-19 who either suffered from complications (death with odds ratio (OR) = 8.81, intensive care unit admissions with OR = 3.54, or assisted ventilation with OR = 4.57) or had been free from those complications, consequently concluding for the first time that chronic periodontitis aggravates mild cases of COVID-19 [38]. This finding clinically supports our abovementioned speculation.

### 4.1. Aspiration of Oral Bacteria May Promote SARS-Cov-2 Infection by Increasing ACE2 Expression on Respiratory Epithelia

Viral and bacterial infections first require binding to specific receptors expressed on host cells for these pathogens. Expression of ACE2, the receptor for SARS-CoV-2, is known to be upregulated by stimuli such as cigarette smoking [39], but upregulation by microbial stimuli has yet to be reported except for ours shown below. When periodontopathic bacteria are aspirated into the lower respiratory tract, the expression of ACE2 can be increased on alveolar and bronchial epithelial cells by stimulation of periodontopathic bacterial cells and periodontal pathogen-derived virulence factors. *Prevotella intermedia*, one of the periodontal pathogens, has been determined to increase the expression of the platelet-activating factor receptor (PAFR) on the alveolar epithelial cells, a receptor for pneumonia-causing bacteria [40]. We have also confirmed that gingipains, the major proteases secreted by *Porphyromonas gingivalis*, not only induce the expression of PAFR but also promote adhesion of *Streptococcus pneumoniae* in an arginine-specific gingipain-dependent manner [41]. Furthermore, gingipains can enhance the expression of sialic acid as an influenza virus receptor by degrading surface proteins of the lower respiratory tract mucosa [42].

**Figure 5:**
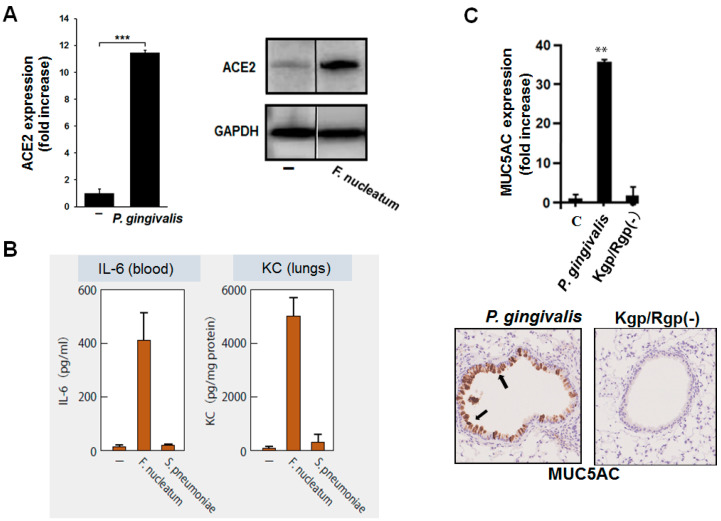
**Induction of ACE2 and production of proinflammatory cytokines by oral bacterial stimulations.** (**A**): Periodontopathic bacterium, *Fusobacterium nucleatum*, in contact with a human alveolar epithelial cell line induced expression of ACE2 mRNA (left panel) and protein (right panel) levels after 48 h. (**B**): Aspiration of *F. nucleatum* in mice induced higher levels of IL-6 and KC (murine IL-8) in the lungs and blood than that of aspiration with *Streptococcus pneumoniae*. (**C**): Culture supernatant of another periodontopathic bacterium, *Porphyromonas gingivalis*, strongly induced the expression of MUC5AC mRNA in mouse lungs (top panel). Protein expression of MUC5AC in the mouse lungs (bottom panel); aspiration of *P. gingivalis* supernatant induced MUC5AC (brown) in the airway epithelium; however, no such effect was observed with gingipain-deficient *P. gingivalis* [Kgp/Rgp (-)]. Student’s *t*-test. **, *p* < 0.01; ***, *p* < 0.001.

From a similar point of view, we have found that *F. nucleatum* induces the expression of ACE2 in alveolar epithelial cells at the mRNA and protein levels [43] (Figure 5A). ACE2 expression was induced more than 20-fold when the bacterial culture supernatant was applied to the epithelial cells. This finding suggests that when periodontal pathogens are aspirated into the lower respiratory tract, they can induce increased ACE2 expression on the epithelial cells of the lower respiratory tract and, thereby, probably promote the infectivity of SARS-CoV-2 to these cells.

This further suggests that contact with periodontal pathogens promotes ACE2 expression on the mucosal epithelia, such as those of the tongue and gingiva, thus increasing the infectivity of SARS-CoV-2 to the oral mucosae.

### 4.2. Aspiration of Oral Bacteria May Stimulate Secretion of Proinflammatory Cytokines and Exacerbate Lung Inflammation

As mentioned above, severe respiratory disorders such as ARDS are one of the main causes of death in patients with COVID-19, and the involvement of a cytokine storm has been specified as a factor causing ARDS. Elevated levels of proinflammatory cytokines and chemokines, such as IL-6, IL-8, and TNF-α, have been strongly associated with mortality, and severely affected individuals are thought to be in a state of excessive inflammation [8,9,10]. Therefore, in addition to antivirals, agents that reduce host inflammation are required as therapeutic agents against severe COVID-19.

We have reported that heat-inactivated periodontal pathogens, such as *F. nucleatum*, *P. gingivalis*, and *P. intermedia*, induce large amounts of proinflammatory cytokines, such as IL-6 and IL-8, from human bronchial, alveolar, and pharyngeal epithelial cell lines [44,45]. Furthermore, these bacteria strongly induce the production of these cytokines not only in cell lines but also in the primary cultures of human respiratory epithelial cells [46]. We have also determined that *P. gingivalis* induces these proinflammatory cytokines via TLR2 on respiratory epithelial cells [45]. In addition, we have shown that the aspiration of heat-inactivated periodontopathic bacteria in mice results in a rapid and strong induction of IL-6 and KC, an isoform of IL-8, in the blood of mice, as well as in their lungs and bronchi [43]; the induced levels of these cytokines were several times higher than those induced by *S. pneumoniae* (Figure 5B). Other researchers have also reported that gingipains can induce pneumonia in mice [47].

In the previous section, we discussed the possibility of aspirated oral bacteria to promote the SARS-CoV-2 infection in relation to PAFR, because platelet-activating factor (PAF) itself increases in gingival crevicular fluid and consequently in saliva in proportion to the degree of inflammation in chronic periodontitis [48].

Therefore, when people with poor oral hygiene aspirate saliva, PAF may bind to PAFR expressed on the mucosa of the lower respiratory tract, naturally raising the proinflammatory status of the lower respiratory tract mucosa.

In fact, we confirmed that PAF induced secretion of proinflammatory cytokines in large amounts by several respiratory mucosal epithelia (article under review).

Therefore, if large amounts of proinflammatory cytokines are induced because of aspirated periodontal pathogens coupled with simultaneously aspirated PAF in patients with COVID-19 who are mildly ill, the infection, coupled with viral-induced pneumonitis, may become severe.

### 4.3. Proteases from Periodontal Pathogens May Promote Viral Infection by Degrading the S Protein of SARS-Cov-2

Influenza virus does not adsorb to host cells unless hemagglutinin (HA) is cleaved into HA1 and HA2. This cleavage occurs by proteolytic action of various proteases secreted by epithelial cells in the human respiratory tract and gastrointestinal tract and also by bacterial proteases of *S. aureus* [49]. We have found that the gingipains degrade HA into HA1 and HA2 (article under review).

During SARS-CoV-2 infection, cleavage of the S protein of SARS-CoV-2 by proteases such as TMSRPP2, as described above, is important for viral attachment to host cells and fusion of host cells with the viral envelope. If the S protein of SARS-CoV-2 is also cleaved by proteases produced by periodontal pathogens, the infectivity of SARS-CoV-2 may be enhanced in patients with chronic periodontitis.

### 4.4. Oral Bacteria May Promote Decreased Respiratory Function and Destruction of Alveoli and Epithelial Barrier

Excessive production of mucin in the respiratory tract not only directly causes excessive sputum production but also physically leads to bronchial narrowing and decreasing respiratory function. We have found that *P. gingivalis* strongly induces the expression of MUC5AC, a core protein of mucin, in a human bronchial epithelial cell line [50]. In addition to some of the detrimental effects of gingipains described above, gingipains may also be involved in this effect. In mouse lung cells, MUC5AC expression and mucin production are strongly induced by the wild-type strain of *P. gingivalis*, but these inductions are not observed with the gingipain-deficient strain [50] (Figure 5C). Therefore, gingipains may induce overproduction of mucin in the human lungs, leading to the narrowing of bronchioles, thereby reducing lung function.

Incidentally, we also found that intratracheally inoculated periodontal pathogens accelerated typical symptoms of COPD, such as destruction of alveoli in COPD model mice induced by elastase instillation (article under review).

**Figure 6:**
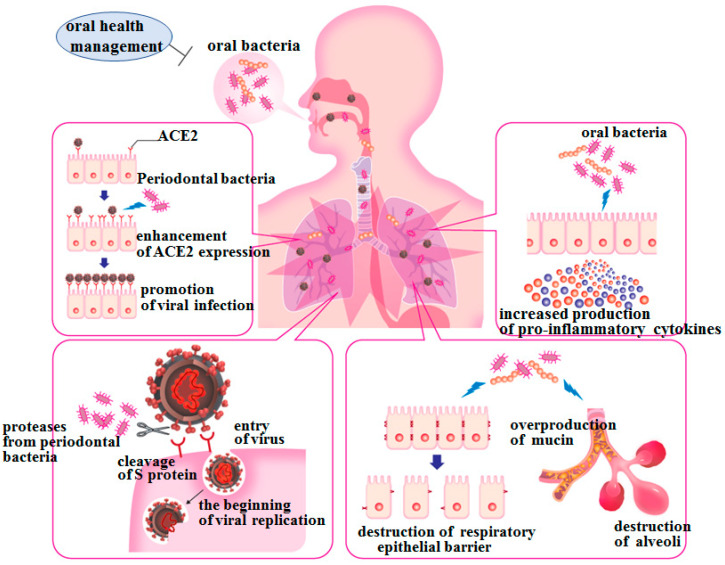
Exacerbation of lower respiratory tract inflammation by stimulation with oral bacteria viewed from the oral cavity-lower respiratory tract axis. The elderly are prone to aspirate oral bacteria along with saliva. In addition, in the current dental situation, the longer the SARS-CoV-2 epidemic continues, the less opportunity there is for professional oral hygiene management, resulting in poor oral hygiene for the subjects with oral health management needs; this is also exacerbated on cases of hospitalization due to COVID-19 attributable to a general shortage of dental professionals in large hospitals. Aspiration of periodontal pathogens and other oral bacteria in patients with COVID-19 induces the expression of ACE2 on the lower respiratory epithelia and the production of proinflammatory cytokines in the lower respiratory tract; this may lead to a “negative spiral” of proinflammatory phenomena in the lower respiratory tract caused by the collaboration between viruses and bacteria. In addition, oral bacteria may induce excessive production of mucin in the lower respiratory tract, resulting in decreased respiratory function, and they may also cause destruction of the epithelial barrier in the alveoli and bronchi. Therefore, oral health management may prevent the aggravation of COVID-19 by suppressing the abovementioned harmful phenomena in patients with COVID-19.

In addition, we confirmed via the use of human cell lines and mice whether oral bacterial stimulation disrupted the barrier function of the alveolar and bronchial epithelia (article under review). These findings suggest that the destruction of respiratory epithelial tight junctions following aspiration of oral bacteria also facilitates the transmission of viruses and pneumonia-causing bacteria between epithelial cells and into subepithelial tissues (Figure 6).

### 4.5. Oral Health Management to Reduce Viral Infections: Prevention of Respiratory Diseases

The longer a patient is hospitalized with COVID-19 and the longer the SARS-CoV-2 pandemic lasts, the less likely they are to receive professional oral health management; these patients are also more likely to develop respiratory inflammation caused by aspiration of oral bacteria because of poorer oral hygiene, even if they are hospitalized because of mild COVID-19, due to the mechanisms described above. However, oral health management administered by dental professionals such as dentists and dental hygienists can reduce mortality from pneumonia and prevent influenza, and multidisciplinary cooperation, represented by medical and dental collaboration, is, therefore, already advancing. In addition, it has been confirmed that oral health management significantly reduces the incidence of ventilator-associated pneumonia in intensive care unit (ICU) patients [51].

There are also many reports that periodontal treatment has improved the condition of patients with COPD and diabetes. Good oral hygiene may lead to the prevention of ACE2 expression and proinflammatory cytokine production in the lower respiratory tract, as well as in the oral cavity. Furthermore, preventing the onset and exacerbation of chronic lung diseases, such as aspiration pneumonia and COPD, through good oral hygiene may decrease susceptibility to COVID-19. This implies that even if infected with SARS-CoV-2, the aggravation of the disease could be prevented by good oral health management.

To better understand COVID-19 from now on, clinical and basic research on the relationship between the oral microbiome and COVID-19, as well as that between oral hygiene and COVID-19 severity should be viewed as important and accessible issues.

### 4.6. Oral Science Expected in the Era of “The New Normal with COVID-19”

To date, most of the research on infectious diseases in the oral cavity has focused only on bacteria as pathogens, and research on the oral cavity and SARS-CoV-2/COVID-19 has been limited due to the need for virus manipulation techniques and dedicated facilities. However, as mentioned above, this kind of research, including that on saliva, is particularly important in understanding the infection risk and mode of transmission and for early detection of infected subjects.

**Figure 7:**
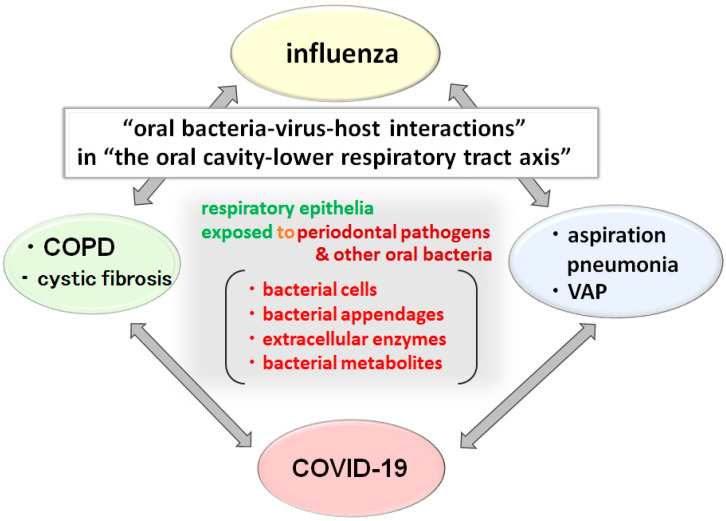
Several “oral bacteria-virus-host interactions” may be involved in giving rise to systemic diseases in “the oral cavity-lower respiratory tract axis” Pneumonia, COPD, influenza, and COVID-19, which are shown to be associated with oral bacteria, involve inflammation of the lungs as the main symptom. In addition, each disease is associated to one another. Therefore, the elucidation of the mechanism of lower respiratory tract inflammation caused by the contact of oral bacteria, extracellular appendages, such as pili and LPS, and exoenzymes with the respiratory epithelium is anticipated. Furthermore, interpreting the effect of the chronic inflammatory state of periodontal disease on systemic chronic inflammation and developing methods to control it is essential. As a result, instead of focusing on “the pathogenicity of a single microorganism” like past studies have, it is essential to examine microbial interactions, such as “oral bacteria-pneumonia-causing bacteria”, “oral bacteria-influenza virus”, and “oral bacteria-SARS-CoV-2”, especially in “the oral cavity-lower respiratory tract axis”.

Further research is expected on the infection mechanism of SARS-CoV-2 in the oral cavity, the detection of SARS-CoV-2 in saliva, and the relationship between SARS-CoV-2 and oral bacteria. Furthermore, the perspective of “oral bacteria-virus-host interactions” (Figure 7) proposed above may foster a better understanding of not only the etiology of oral diseases but also of systemic diseases caused due to the oral microbiota especially in “the oral cavity-lower respiratory tract axis”. This may lead to the development of new treatment and prevention methods for these diseases and to the expansion of the fields in which dental care and dental research are involved.

## 5. Conclusions

Through the mechanisms discussed above, it can be inferred that periodontal pathogens and other oral bacteria have a degree of influence on the development of COVID-19. However, due to “refraining from dental visits” caused by the spread of COVID-19, an increase in deterioration in oral hygiene and the condition of teeth and periodontal tissues is also presumed to have occurred; additionally, damage to periodontal tissues due to chronic periodontitis may enable the virus to invade the human body more easily through the periodontium affected by chronic periodontitis. Moreover, periodontal pathogens and their virulence factors, such as endotoxin, can enter blood vessels and, in combination with SARS-CoV-2 infection, aggravate COVID-19.

The long-awaited vaccines to prevent SARS-CoV-2 infection and COVID-19 aggravation have finally been completed, and the number of people who have received the vaccine is increasing daily. However, the distribution of the vaccine remains uneven among countries and regions, and the amount of vaccine produced remains insufficient. So far, a sovereign remedy for COVID-19 has proved elusive, the long-lasting effects of the immunity induced by the newly developed vaccines remains unclear, and there is an undeniable possibility that mutant strains could negate the effectiveness of these vaccines [52]; thus, it is not an exaggeration to state that oral health management, as well as the wearing of masks and the encouragement of hand washing and disinfection, is fundamental to COVID-19 control, even after the vaccine has been globally disseminated. Since perioperative oral health management can prevent post-operative complications such as pneumonia in patients who have undergone surgery, there is a great possibility that the promotion of oral health management will lead to the alleviation of emergency medical care and the reduction in medical costs in many countries. Finally, even if humans can eradicate SARS-CoV-2, there remains the possibility that mutant coronavirus strains that occur in wild animals will infect humans again. In other words, the possibility of a new coronavirus outbreak is high.

As mentioned above, the elderly are particularly susceptible to lower respiratory tract infections, and in countries where the population is aging, measures against infectious diseases, including viral infections, will become even more important. In addition, there is another point of view that should not be overlooked. An in vitro experiment using a respiratory epithelial cell line infected with SARS-CoV-2 indicated that infection with SARS-CoV-2 not only reduces the activity of the epithelial cilia but also induces the shedding of ciliated epithelial cells [53]. This strongly suggests that the ciliated respiratory epithelium infected by SARS-CoV-2 no longer has an ability to exclude foreign substances, which consequently indicates that the oral bacteria aspirated into the lower respiratory tract will never be eliminated for the duration of COVID-19 infection. In other words, even in mild COVID-19 cases, there may be a proinflammatory status in the lower respiratory tract stimulated by aspirated oral bacteria, irrespective of their vitality. It should be noted that this is completely contrary to the recently established therapeutic strategy for patients with severe COVID-19 by suppression of ARDS and may have led mildly infected patients with poor oral hygiene into a so-called “negative spiral”. It should also be noted that, as mentioned above, the recently published retrospective clinical study has showed that patients who are affected with both COVID-19 and chronic periodontitis suffer from complications (death, ICU admissions, or assisted ventilation) ascribable to aggravation of mild COVID-19 [38].

Provided that “all people who are currently refraining from seeing a dentist” resume dental visits and treatment, dentists may be able to detect viral infections at an early stage based on the interview with and the oral findings from the patient. This offers the opportunity to provide oral health management by controlling oral hygiene and managing oral functions, including both swallowing and eating, which may well protect those patients from aggravation of COVID-19.

The theory introduced above, i.e., poor oral hygiene leading to chronic periodontitis; aspiration even of dead oral bacteria, including periodontal pathogens; and the resulting pro-inflammatory status of the lower respiratory tract that are associated with the susceptibility of mild COVID-19 deteriorating to a more severe disease, must await the results from large clinical randomized controlled trials. However, it is desirable that all medical professionals equally understand the viewpoint of “oral bacteria-virus-host interaction” and deepen their understanding of systemic diseases affected by oral microflora. Medical and dental cooperation can then promptly respond to not only the current COVID-19 pandemic but also to future pandemics that we are yet to encounter.

## Figures and Tables

**Table 1:** Characteristics of coronavirus that infects humans.

	SARS-C0V-2	SARS-C0V-1	MAERS-CoV	HCoV-229E, OC43, NL63&KHU-1
Name of infection	COVID-19 (coronavirus disease 2019)	SARS (severe acute respiratory syndrome)	MERS (Middle East respiratory syndrome)	Common cold syndrome
Year of outbreak	2019 to present	2002–2003	2012 to present	Annually
Endemic areas	worldwide	Mainland China & Hong Kong	Middle East	worldwide
Original host	Horseshoe bat?	Horseshoe bat	Arabian camel	Human
Number of infected people	169 million (at the end of May, 2021)	8098	2589 (at the end of May, 2021)	7 billion
Number of deaths	3.53 million	774	940	Uncertain
Incubation period	1–2 weeks (mostly 5–6 days)	2–10 days	2–2 weeks	2–4 days

## Data Availability

No new data were created or analyzed in this study. Data sharing is not applicable to this article.
